# Return of individual genomic research results within the PRAEGNANT multicenter registry study

**DOI:** 10.1007/s10549-022-06795-x

**Published:** 2022-11-21

**Authors:** Hanna Huebner, Matthias Ruebner, Christian Kurbacher, Peyman Hadji, Andreas D. Hartkopf, Michael P. Lux, Jens Huober, Sabrina Uhrig, Florin-Andrei Taran, Friedrich Overkamp, Hans Tesch, Lothar Häberle, Diana Lüftner, Markus Wallwiener, Volkmar Müller, Matthias W. Beckmann, Alexander Hein, Erik Belleville, Michael Untch, Wolfgang Janni, Tanja N. Fehm, Hans-Christian Kolberg, Diethelm Wallwiener, Sara Y. Brucker, Andreas Schneeweiss, Johannes Ettl, Peter A. Fasching, Laura L. Michel

**Affiliations:** 1 Department of Gynecology and Obstetrics, Comprehensive Cancer Center Erlangen-EMN, University Hospital Erlangen, Friedrich-Alexander-Universität Erlangen-Nürnberg (FAU), Erlangen, Germany; 2 Department of Gynecology and Obstetrics, Medizinisches Zentrum Bonn Friedensplatz, Bonn, Germany; 3 Frankfurt Center for Bone Health, 60313 Frankfurt, Germany; 4 Department of Obstetrics and Gynecology, University of Tübingen, Tübingen, Germany; 5 Klinik Für Gynäkologie und Geburtshilfe Frauenklinik St. Louise, St. Josefs-Krankenhaus, Salzkotten, Kooperatives Brustzentrum Paderborn, Paderborn, Germany; 6 Department of Gynecology and Obstetrics, Ulm University Hospital, Ulm, Germany; 7 OncoConsult Overkamp GmbH, Berlin, Germany; 8 Oncology Practice at Bethanien Hospital Frankfurt, Frankfurt, Germany; 9 Biostatistics Unit, Department of Gynecology and Obstetrics, University Hospital Erlangen, Erlangen, Germany; 10 Immanuel Hospital Märkische Schweiz, Buckow, Germany; 11 Department of Obstetrics and Gynecology, University of Heidelberg, Heidelberg, Germany; 12 Department of Gynecology, Hamburg-Eppendorf University Medical Center, Hamburg, Germany; 13 ClinSol, GmbH & Co KG, Würzburg, Germany; 14 Department of Gynecology and Obstetrics, Helios Clinics Berlin Buch, Berlin, Germany; 15 Department of Gynecology and Obstetrics, University Hospital of Düsseldorf, Düsseldorf, Germany; 16 Marienhospital Bottrop, Bottrop, Germany; 17 National Center for Tumor Diseases, University Hospital and German Cancer Research Center, Heidelberg, Germany; 18 Department of Obstetrics and Gynecology, Klinikum rechts der Isar, Technical University of Munich, Munich, Germany; 19 Immanuel Campus Rüdersdorf/Medical University of Brandenburg, Brandenburg, Germany

**Keywords:** Genetic testing, Research results, Return of results, Incidental findings, Breast cancer

## Abstract

**Purpose:**

The PRAEGNANT study is a registry study for metastatic breast cancer patients, focusing on biomarker detection. Recently, within this study, genetic alterations in 37 breast cancer predisposition genes were analyzed and genetic findings were detected for 396 participants. The aim of this project was to return genetic results to the physicians and to analyze actions taken (e.g., disclosure of results to patients, validation of results, clinical impact, and impact on the patient’s quality of life) using a questionnaire.

**Methods:**

235 questionnaires were sent out to the study centers, with each questionnaire representing one patient with a genetic finding. The questionnaire consisted of twelve questions in the German language, referring to the disclosure of results, validation of test results, and their impact on treatment decisions and on the patient’s quality of life.

**Results:**

135 (57.5%) questionnaires were completed. Of these, 46 (34.1%) stated that results were returned to the patients. In 80.0% (*N* = 36) of cases where results were returned, the patient had not been aware of the finding previously. For 27 patients (64.3%), genetic findings had not been validated beforehand. All validation procedures (*N* = 15) were covered by the patients’ health insurance. For 11 (25.0%) patients, physicians reported that the research results influenced current or future decision-making on treatment, and for 37.8% (*N* = 17) the results influenced whether family members will be genetically tested.

**Conclusion:**

This study provides novel insights into the return of research results and into clinical and personal benefits of disclosure of genetic findings within a German registry.

**Supplementary Information:**

The online version contains supplementary material available at 10.1007/s10549-022-06795-x.

## Introduction

Many clinical trials and translational research projects involve genomic analysis of both germline and somatic DNA. In particular, the continuous fall in the cost of sequencing and the availability of quick and affordable methods for analysis of genomic alterations are leading to a steady increase in the generation of genomic data. In addition, the giving of broad consent for the provision of biosamples (e.g., blood or tissue) for central biobank units is becoming more popular, which means we can expect a further increase in the number of samples available for genomic analyses. These biobanks support numerous additional associated studies and research projects, so are likely to generate a further steady rise in available genomic data.

The source of such genomic data can be whole genome or exome sequencing or specific analysis of certain gene panels associated with the risk of disease. Whole genome and exome sequencing in particular have an increased probability of incidental genetic findings which may be relevant to issues such as treatment decisions, genetic testing of relatives or enhanced early diagnosis and disease prevention programs. Thus, returning individual genetic research results to patients or study participants may generate clinical and personal benefits. However, knowledge about such genetic findings can also result in increased anxiety, unnecessary follow-up testing or therapeutic misperceptions, and not everyone wishes to know about alterations detected in their case [1–4].

This said, the vast majority of patients who participate in clinical trials are in favor of receiving results from these trials; in addition, patients’ willingness to participate in studies increases when research results are returned to them [5, 6]. However, return of genomic research results demands substantial financial resources and time for the identification of relevant mutations, the interpretation of their clinical relevance, and genetic counseling [7, 8]. Further, these processes require a defined and standardized workflow, especially if research results have to be communicated from a central research unit to multiple study centers or treating physicians.

For breast cancer patients, genomic data are becoming increasingly important due to the rising number of therapeutic approaches that rely on genetic alterations. For example, alterations in the breast cancer risk genes BRCA1 and 2 are biomarkers for treatment with poly (ADP-ribose) polymerase (PARP) inhibitors. Similarly, other genes associated with BRCAness or involved in DNA repair by homologous recombination (e.g., *PALB2, ATM, CHEK2, RAD51, MSH2,* and *BARD1*) are also assumed to be good indicators for clinical response to PARP inhibitor therapy [9–11]. Recently, 21 candidate genes, including *BRCA1* and *2*, were associated with breast cancer risk in a large case–control study [12]. The presence of one or more of those germline alterations could be an indicator for genetic testing of relatives and could be or become clinically relevant with regard to targeted therapeutic options—especially for metastatic breast cancer patients, who have a poor prognosis and limited treatment options.

Currently, genotyping of certain breast cancer risk genes is only performed in a number of defined situations within the clinical routine setting. For example, eligibility criteria for germline genetic testing in Germany include breast cancer diagnosis before the age of 46 years, bilateral breast cancer before the age of 51 years, more than two breast cancer cases in the family, a first-degree relative with ovarian cancer, and a male relative with breast cancer. In addition, genetic testing may be performed if it is of relevance to further decision-making on treatment (e.g., PARP inhibitor therapy). However, a certain number of patients not eligible for genetic testing might also harbor mutations in genes related to the risk of breast or other cancers, which may be of relevance to clinical decisions and familial risk. Thus, the returning of genetic research results to patients with breast cancer might be of particular benefit in patients who are not eligible for routine genetic testing.

The aim of this study was to return individual research results from panel genetic testing of 37 cancer predisposition genes to treating physicians within the multicenter PRAEGNANT registry study and to analyze actions taken after the return of results (e.g., disclosure of results to patient, validation of results, clinical impact, and impact on the patient’s quality of life) using a questionnaire.

## Materials and methods

### The PRAEGNANT research network

The PRAEGNANT study *(*Prospective Academic Translational Research Network for the Optimization of Oncological Health Care Quality in the Adjuvant and Advanced/Metastatic Setting; NCT02338167*)* is an ongoing registry study for advanced, metastatic breast cancer patients [13]. Patients can be included in the study at any time and stage of disease. All participants signed an informed consent form before joining the study. Patients with metastatic or locally advanced, inoperable disease proven by clinical measures (i.e., standard imaging) are eligible for study inclusion. As part of the study, blood samples and formalin-fixed paraffin-embedded tissue samples are collected and stored in the central biobank unit [13]. The primary objective of the PRAEGNANT study is to investigate biomarkers for breast cancer disease and progression. Further aims of the study are to assess treatment patterns and to identify patients who may be eligible for inclusion in clinical trials [13–16]. The study was approved by the relevant ethics committees (Ethical approval number: 234/2014BO1, first approval on June 17, 2014, approval of Amendment 1 on June 11, 2015, approval of Amendment 2 on March 18, 2019; Ethics Committee of the Medical Faculty, University of Tübingen, Tübingen, Germany).

### Germline genetic testing

Between July 2014 and March 2018, 2647 metastatic breast cancer patients were enrolled in the PRAEGNANT study. EDTA blood samples were collected from each patient and germline DNA was isolated and tested for variations within 37 cancer predisposition genes. A custom amplicon-based QIAseq panel (QIAGEN, Hilden, Germany) was used as previously described [17]. The following genes were analyzed within the stated panel: *APC, ATM, BARD1, BLM, BRCA1, BRCA2, BRIP1, CDH1, CDKN2A, CHEK2, EPCAM, ERCC2, ERCC3, FANCC, FANCM, KRAS, MEN1, MLH1, MRE11A, MSH2, MSH6, MUTYH, NBN, NF1, PALB2, PMS2, PPM1D, PRSS1, PTEN, RAD50, RAD51C, RAD51D, RECQL, RINT1, SLX4, TP53*, and *XRCC2.* High-quality sequencing data were obtained from 2595 PRAEGNANT patients. Sequencing data and associations between mutation status and tumor characteristics, progression-free survival, and overall survival were published recently [18]*.*

### Return of research results

Genomic research results were merged with PRAEGNANT patient IDs in the central data management unit of the PRAEGNANT registry study. Research results were returned to the relevant study site and treating physician. Each letter to the treating physician stated that, due to the pseudonymization of patient and biomaterial data, the correct assignment of test results could not be guaranteed and that there are currently no data available regarding the sensitivity of the testing method used. Further, treating physicians were advised to validate any detected mutations before acting on the findings in terms of treatment decisions.

### Questionnaire on return of research results

After receiving the returned research results, treating physicians were asked to complete a questionnaire. The questionnaire consisted of twelve questions in the German language, referring to the disclosure of results to the relevant patient, validation of test results, and their impact on treatment decisions treatment and on the patient’s quality of life. A translated version of the questionnaire can be found in Supplementary File 1. All questions were single-choice questions, apart from the second question, “*If you did NOT inform the patient, what were your reasons for this?”*, which was a multiple-choice question (Table 1). Completed questionnaires were returned to the central biobank unit of the PRAEGNANT registry study for analysis. Results are presented in numbers and percentages. Data were analyzed using IBM SPSS statistics Version 24.

**Table 1:** Return of research results

		*N* (%)
Did you return the results to the patient?	No	89 (65.9)
	Yes	46 (34.1)
	Total	135 (100.0)
Reasons for not returning the results^a^ (multiple-choice question)	Patient did not agree to receiving results upon study inclusion	8 (9.0)
	Patient did not agree to receiving results after consultation	1 (1.1)
	The result(s) have no relevance to the patient or their family	34 (38.2)
	I do not trust the results	0 (0.0)
	I do not understand the results	0 (0.0)
	Other reason	57 (64.0)
Total		100 (112.4^*^)

^a^Group: Did you return the results to the patient? = No

*The question was a multiple-choice question. Therefore, the percentage is greater than 100%

## Results

### Return of research results

**Fig. 1:**
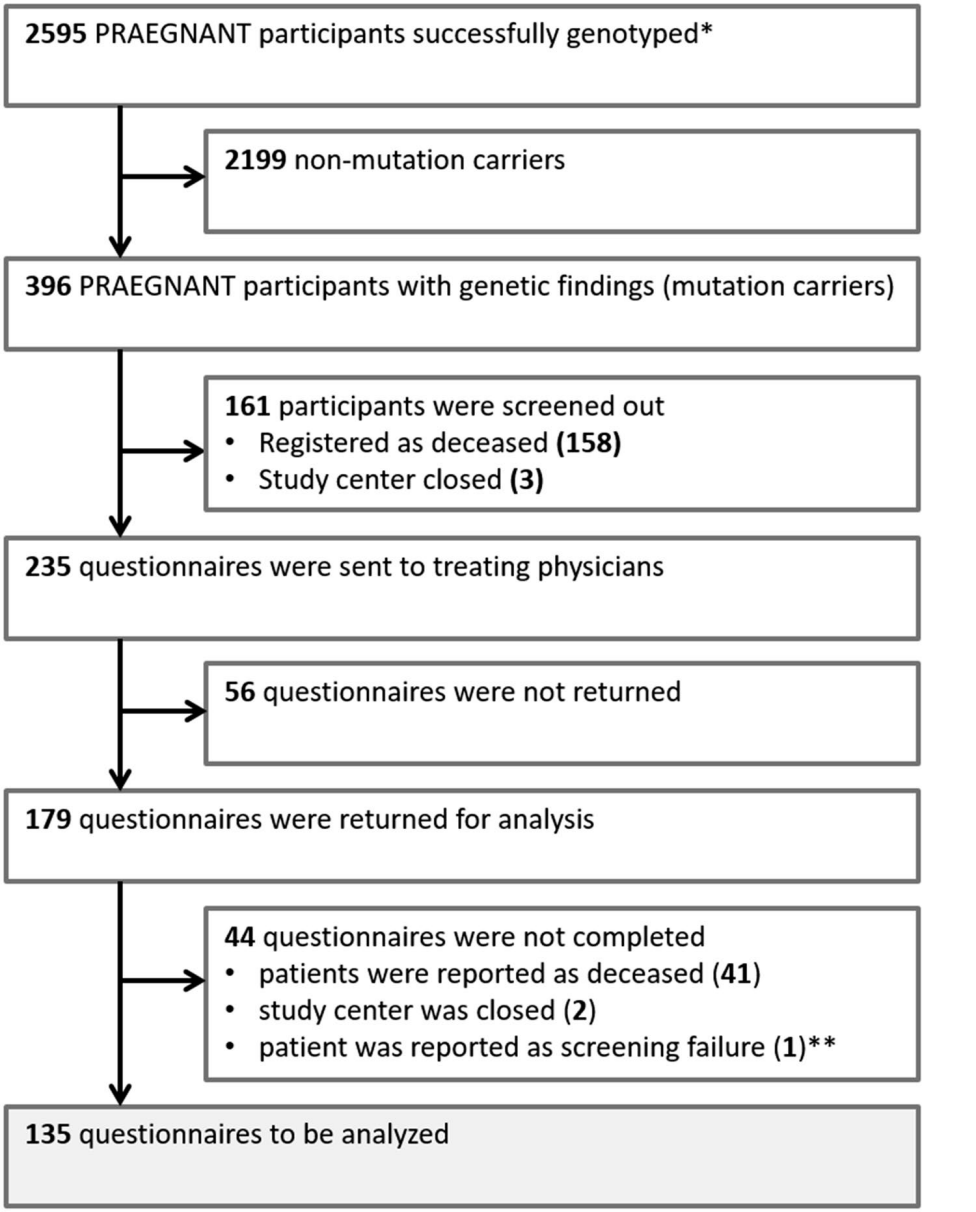
Patient flowchart. * Genotyping results were published in Fasching et al. [18]. ** Deaths of patients and screening failures were not documented in eCRF before questionnaires were sent out

Genomic research results from a study published recently [18] were available from 2595 metastatic breast cancer patients enrolled in the PRAEGNANT registry (Fig. 1). Genotyping results for each patient were sent to the patient’s treating physician at each study center in June 2019. Of these patients, 2,199 were non-mutation carriers, while 396 PRAEGNANT participants had at least one genetic finding (Fig. 1). Questionnaires on the return of research results, validation of results, and the impact of genetic findings were sent out to the treating physicians in March 2020. Of 396 eligible patients, 161 were excluded due to being registered as deceased or due to the study center being closed at the time of the questionnaires’ distribution (Fig. 1). Thus, 235 questionnaires were sent out to the study centers, with each questionnaire representing one patient with a genetic finding. As of January 2022, 179 (73.9%) questionnaires had been returned for analysis, of which 41 had not been completed due to the patient being deceased or due to screening failure (Fig. 1). Thus, 135 questionnaires were analyzed.

**Fig. 2:**
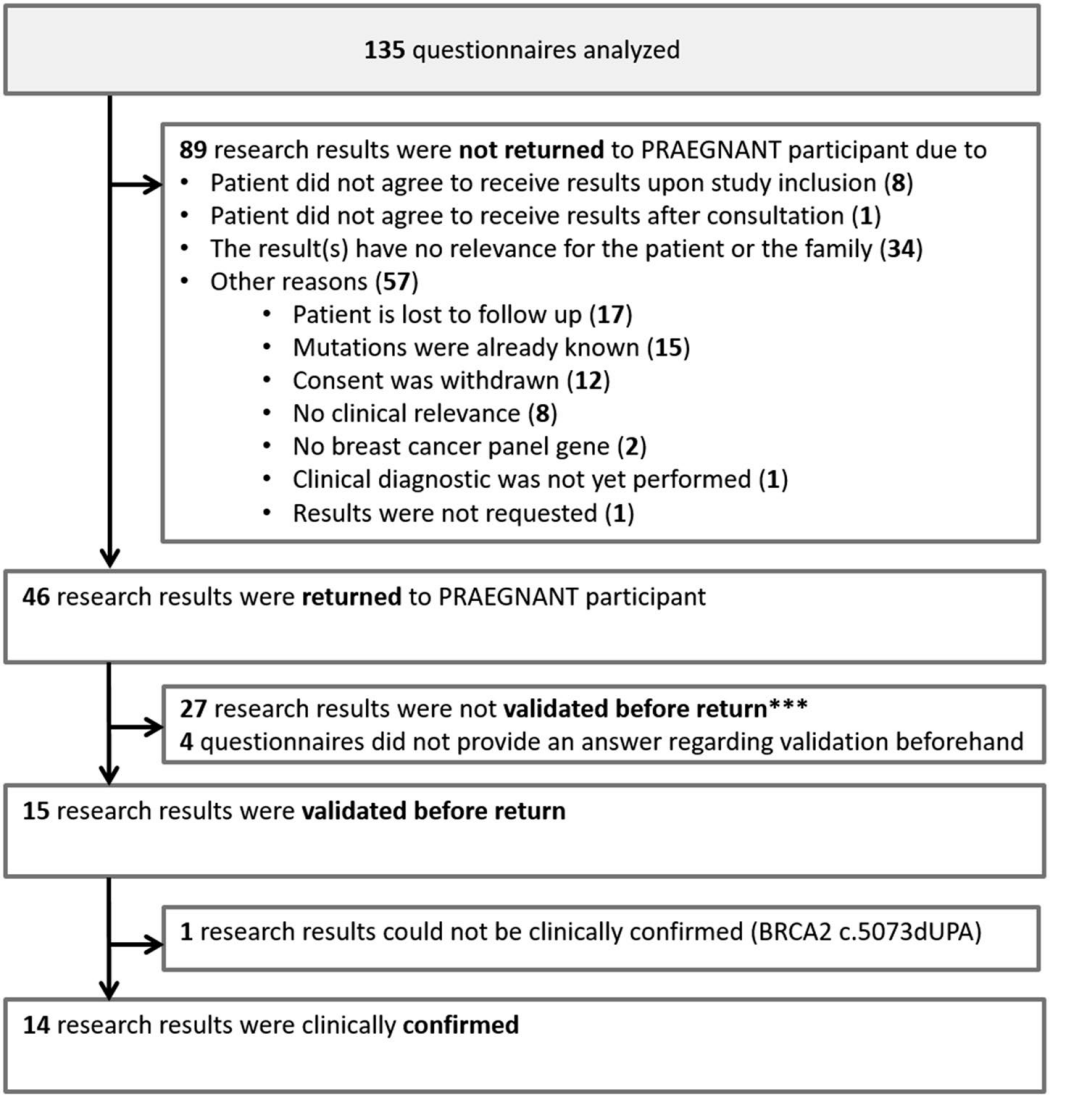
Return and validation of research results. *** For four patients, validation after return of results was reported

89 (65.9%) questionnaires reported that research results were not returned to the patient, while 46 (34.1%) stated that results were returned (Table 1). The most commonly cited reasons for not returning the results were a lack of relevance of the results to the patient or the patient’s family (38.2%) and “[an]other reason,” that is, a reason not specified in the question (64.0%) (Table 1). Other reasons included, for example, loss of follow-up (*N*=17), the patient already being aware of the mutations (*N*=15), withdrawal of consent (*N*=12), and lack of clinical relevance (*N*=8) (Fig. 2, Supplementary Table 1). None of the treating physicians stated that they did not trust or understand the results (Table 1).

In 80.0% of cases (*N*=36) in which the results were returned to the patient, none of the genetic findings had been known by the patient beforehand (Table 2). Of those cases in which genetic findings were not returned to the patient, 49.0% (*N*=24) did not know about any of the genetic alterations detected (Table 2). For 19 of the 24 (79.2%) cases in which the mutations detected had not been known previously, but also not returned to the patient, physicians stated that the result(s) were not returned due to a lack of relevance to the patient or the family (data not shown).

**Table 2:** Knowledge about mutations

		Total *N* (%)	Return of results = YES, *N* (%)^b^	Return of results = NO, *N* (%)^a^
Were the germline mutations detected already known to the patient?	No, none	60 (63.8%)	36 (80.0%)	24 (49.0%)
	Yes, all	27 (28.7%)	6 (13.3%)	21 (42.9%)
	Yes, but not all	7 (7.4%)	3 (6.7%)	4 (8.2%)
	Total	94 (100.0%)	45 (100.0%)	49 (100.0%)
	Missing	41	1	40

^a^Group: Did you return the results to the patient? = No

^b^Group: Did you return the results to the patient? = Yes

### Validation of research results

Where treating physicians returned research results to the patient, they did not validate these genetic findings beforehand in 64.3% (*N*=27) of cases (Table 3, Fig. 2). In 31.0% (*N*=13) of cases, all genetic findings were validated before return of results; in 4.8% (*N*=2) of cases, results were validated, but not all of them (Table 3). In 4 cases, research results were reported as validated, but only after return of genetic findings (data not shown). Of those results that were validated before return to the patient, all mutations detected were validated in 60.0% of cases, only mutations selected by the physician in 13.3%, and only mutations selected by a geneticist in 26.7% (Table 3). In one case, the validated results were not in line with the genetic findings attained in the PRAEGNANT study; all other validated cases (93.3%) were in line with PRAEGNANT test results (Table 3). In the one finding with failed validation of results, the research results found a *BRCA2* mutation (c.5073dupA); validation, however, revealed a wild-type BRCA2 sequence. Each of the reported validations of research results was covered by the patient’s health insurance (*N*=15; 100%; Table 3).

**Table 3:** Validation of research results

		*N* (%)
If the results were returned to the patient, were they validated beforehand^a^	No, none	27 (64.3%)
	Yes, all	13 (31.0%)
	Yes, but not all	2 (4.8%)
	Total	42 (100.0%)
	Missing	3
If results were validated, which mutations were analyzed?^a,b^	All mutations detected	9 (60.0%)
	Only mutations selected by the physician	2 (13.3%)
	Only mutations selected by the geneticist	4 (26.7%)
	Total	15 (100.0%)
If results were validated, were they in line with the PRAEGNANT test results?^a,b^	No, not all	1 (6.7%)
	Yes, all	14 (93.3%)
	Total	15 (100.0%)
Did the patient’s health insurance cover the cost of validation?^a,b^	No	0 (0.0%)
	Yes	15 (100.0%)
	Total	15 (100.0%)

^a^Group: Did you return the results to the patient? = Yes

^b^Group: Were the results validated before being returned? = Yes

### Impact of research results

Where research results were returned to the patients, treating physicians were asked whether these results had influenced decision-making on treatment, genetic testing of family members, or the patient’s quality of life. In 11 (25.0%) cases, physicians reported that the PRAEGNANT research results had influenced or would influence current or future treatment decisions (Table 4); and in 37.8% (*N*=17) of cases, respondents stated that results had influenced decisions on whether family members will be genetically tested (Table 4). In 19 (46.3%) cases, physicians believed the patients did benefit from the PRAEGNANT test results. When asked whether they thought that the PRAEGNANT test results influenced the patient’s quality of life, 36 (80.0%) responded that they did not think so, 7 (15.6%) believe their quality of life was influenced positively, and 2 (4.4%) perceived a negative influence (Table 4). In 9 of the 11 cases where physicians stated that treatment decisions were or will be influenced, eligibility for PARP inhibitor therapy was the reason given (Table 5). Respondents explained their view of a positive influence on patient quality of life with access to PARP inhibitor therapy, additional treatment options, or cancer screening for children (Table 5). One physician who reported a negative impact on the patient’s quality of life cited the patient’s anxiety about passing on the mutation (Table 5).

**Table 4:** Impact of research results

		*N* (%)
Did the results influence current or future treatment decisions?^a^	No	33 (75.0%)
	Yes	11 (25.0%)
	Total	44 (100.0%)
	Missing	2
Did the results influence whether family members will be genetically tested?^a^	No	13 (28.9%)
	Yes	17 (37.8%)
	I don't know	15 (33.3%)
	Total	45 (100.0%)
	Missing	1
Do you think the patient benefited from the PRAEGNANT test results?^a^	No	22 (53.7%)
	Yes	19 (46.3%)
	Total	41 (100.0%)
	Missing	5
Do you think the PRAEGNANT test results will influence the patient's quality of life?^a^	No	36 (80.0%)
	Yes, positively	7 (15.6%)
	Yes, negatively	2 (4.4%)
	Total	45 (100.0%)
	Missing	1

^a^Group: Did you return the results to the patient? = Yes

**Table 5:** Implications of genetic results in patients to whom results were returned (*N* = 42)

Age range at diagnosis (yrs)					Clinical implications						
		Mol. subtype	Gene	Known mutation^a^	Influence on treatment?^b^	Reason	Influence on testing of relatives?^c^	Benefit?	Reason^d^	Influence QoLe	Reason
18–50		Lum A	BARD1	No	No		I don't know	Yes	Exclusion of a highly penetrating mutation	No	
18–50		Lum B, HER2 −	BRCA1	No	Yes	PARPi therapy, prophylactic adnexectomy	Yes	Yes	prophylactic adnexectomy, olaparib as additional therapy option, testing of relatives	Yes, +	PARPi therapy
18–50		Lum B, HER2 −	BARD1	No	No		I don't know		Exclusion of a highly penetrating mutation	No	
18–50		–	BRCA1	No	Yes		Yes	Yes		Yes, +	Additional treatment option
18–50		Lum A	BRCA1	Yes, all	No		No	No		No	
18–50		–	RINT1	No	No		No	No		No	
18–50	HER2 +		ATM	No	No		I don't know	No		Yes, −	
18–50			BARD1	No	No		No	No		No	
18–50		–	ATM	No	No		I don't know	No		No	
18–50		HER2 +	ERCC2	No	No		I don't know	No		No	
18–50		TNBC	BRCA1; FANCC	Yes, all	No		No	No		No	
18–50		Lum A	BRCA2	Yes, but not all	No		Yes	No		No	
18–50		HER2 +	TP53	Yes, all	No		No	No		No	
18–50		TNBC	BRCA1	No	Yes	PD and PARPi therapy	Yes	Yes		No	
18–50		HER2 +	CHEK2		No		Yes	Yes	Cancer screening for children	Yes, +	Cancer screening for children
18–50		TNBC	BRCA2	Yes, all	Yes	PARPi therapy	Yes	Yes	PARPi therapy	Yes, +	
18–50		HER2 +	TP53	No	No		I don't know			No	
18–50		Lum A	BRCA2	No	No		No	No		No	
18–50		–	BRIP1	No	No		I don't know	No		No	
18–50		Lum A	CHEK2	No	No		No	No		No	
18–50		–	ATM	No	No		Yes	Yes	Testing of relatives	No	
18–50		Lum A	ATM	No	No		Yes	Yes	Testing of relatives	No	
18–50		Lum A	BRCA2	Yes, all	No		No	No		No	
18–50		HER2 +	TP53	No	No		No			No	
18–50		HER2 +	BRCA2	No	Yes	PARPi therapy	Yes	No	Olaparib attempt without effect (very advanced situation)	No	
18–50		HER2 +	BRCA2	No	No	HER2 +	Yes	No	No change in therapy but another family member tested + !	No	
18–50		–	BRCA2	No	Yes	PARPi therapy		Yes	Explanation for disease		
18–50		MSH6		No			No	No		No	
18–50		BRCA2		No	Yes	PARPi therapy	No	Yes		Yes, +	PARPi therapy
> 50		Lum A	CHEK2	No	No		I don't know	Yes	Exclusion of a highly penetrating mutation	No	
> 50		HER2 +	BRCA2	No	Yes	PARPi therapy	Yes	Yes	PARPi therapy, testing of relatives	Yes, +	PARPi therapy
> 50		Lum B, HER2 −	ATM	No	No		I don't know	Yes	Exclusion of a highly penetrating mutation, relevance for children	No	
> 50		HER2 +	BRCA1	No	Yes	PARPi therapy	Yes	Yes	PARPi therapy, testing of relatives	Yes, +	PARPi therapy
> 50		Lum A	ERCC3	No	No		I don't know			No	
> 50		Lum A	PMS2	No	No		I don't know	No		No	
> 50		HER2 +	NF1	No	No		I don't know	Yes	Early diagnosis	Yes, −	Anxious about passing on the mutation
> 50		Lum A	TP53	No			I don't know	Yes	Testing of relatives	No	
> 50		Lum A	CHEK2	No	Yes	PARPi therapy	Yes	No		No	
> 50		HER2 +	RAD50	No	No		No	No		No	
> 50		Lum B, HER2 −	BRCA2; CHEK2	Yes, but not all	No		I don't know	No		No	
> 50		Lum A	BRCA2	No	No		Yes	Yes	Testing of children	No	
> 50		–	MUTYH	No	No		No			No	
> 50		HER2 +	BRCA2	No	No	Was recommended, patient refused	I don't know	No	Patient could not decide upon validation of results	No	Patient could not decide upon validation of results
> 50		BRCA1	TNBC	Yes, but not all	Yes		Yes	Yes	Impact on treatment decisions	No	
> 50		BRCA1	Luminal A	Yes, all	No		Yes	No		No	

^a^Were the detected germline mutations already known by the patient?

^b^Did the results influence current or future treatment decisions?

^c^Did the results influence whether family members will be genetically tested?

^d^Do you think the patient benefited from the PRAEGNANT test results?

^e^Do you think the PRAEGNANT test results will affect the patient's quality of life?

## Discussion

In this large cohort of metastatic breast cancer patients who are enrolled in the PRAEGNANT registry study, we recently identified 396 participants with genetic findings in one or more breast cancer risk genes [18]. For 235 breast cancer patients, genetic research results and the associated questionnaires regarding return of results were sent out to their respective treating physicians. 135 completed questionnaires were analyzed. Of these, 63.8% (*N*=60) reported that the patients had not previously known about the mutations detected. This figure represents 2.3% of the complete cohort of PRAEGNANT participants successfully genotyped (*N*=2595) [18]. In 11 cases, the findings influenced current or future treatment decisions, while in 17 cases, the results influenced the decisions on whether family members will be genetically tested.

The questionnaires revealed that 65.9% of research results were not passed on to the patients. The most commonly cited reason for physicians not returning the results was that they did not perceive these results to have any relevance to the patient or the patient’s family. Alterations which physicians categorized as “having no relevance to the patient or their family,” despite the patient being unaware of them, occurred, for example, in *MSH6* and *PMS2* genes. The association of such mutations with breast cancer risk is still a topic of debate among experts in the field [19–21]. However, although those genes may still harbor high uncertainty regarding breast cancer risk, they are part of the mismatch repair machinery (*MSH2, MSH6, MLH1, PMS2*) associated with Lynch syndrome, and loss of expression is accompanied by microsatellite instability [22]. A small number of trials indicate that cancers with microsatellite instability show a good response to immune checkpoint therapy (e.g., anti-PD1 inhibitors), particularly in patients for whom standard therapeutic approaches failed [22, 23]. This shows that even though the clinical significance of these mutations is still unclear, future studies and novel targeted therapeutic approaches may lead to the discovery of novel biomarkers, and such knowledge about these germline alterations may thus gain importance in the future.

In 2013 and 2016 (updated version), the American College of Medical Genetics and Genomics (ACMG) published a policy statement on recommendations for reporting of incidental genomic findings [24, 25]. These ACMG recommendations state that mutations found within genes from the so-called “minimum list” (including 59 medically actionable genes) could likely be of medical benefit to patients and their families and thus should be reported to the patient’s treating physician [24, 25]. This minimum list includes genes associated with a phenotype, such as hereditary breast or ovarian cancer (*BRCA1* and *2*), Li–Fraumeni syndrome (*TP53*), or Lynch syndrome (*MLH1, MSH2, MSH6, PMS2*), besides others [24, 25]. Within our PRAEGNANT study, of those mutations that were not returned to the patient, not known by the patient and classified as “not relevant” by the physician, 7 of 21 findings (33%) appear on the minimum list (*MUTYH*, *TP53, PMS2, MSH6*) (Supplementary Table 1).

A recent evaluation of interviews with healthcare providers revealed that physicians’ knowledge gaps in genomics are one of the key challenges in returning results of high quality [26]. Suggestions for improvement included standardized consultation notes, educational conferences, and a panel of experts for additional referrals [26]. However, it is necessary here to take into account the fact that research results with high uncertainty as regard their clinical relevance may be associated with considerably time-consuming patient–physician discussions and with the burdensome time and financial cost of involving a geneticist for additional professional expertise [27, 28].

Earlier evaluations of the experience and attitudes of study participants and patients revealed that their experience of receiving returned results was fairly positive, while attitudes among the group of healthcare professionals and researchers were less positive and more cautious [29]. The main concern of professionals was associated with the uncertainty of results and the blurring of basic research and clinical care [29–31]. This concern could result in more paternalistic decision-making. Paternalism is an attitude associated with actions taken by a physician with the intent of providing the best care for the patient, but without the patient’s consent. In our study, some of the results were not returned even though the patients opted in to the receipt of relevant genetic research results. In general, shared decision-making is the preferred way for patients and clinicians to jointly make healthcare decisions. However, studies have shown that, in day-to-day practice, paternalistic decision-making occurs more often than expected [30]. In particular, junior physicians seem to be uncertain about implementing shared decision-making [30, 32]. This may be due to physicians being presented, particularly during medical education, as the ones responsible for clinical decisions and for convincing patients to follow these choices [32–34]. Patients often consider genetic findings to be equivalent to other medical test results. In contrast, healthcare professionals may strongly associate genetic findings with the concept of “genetic exceptionalism” [35], which holds that genetic information is a special category of medical knowledge data which has to be handled with particular caution. This special handling of genetic data has even been codified in the scientific guidelines of the German Society of Human Genetics (GfH) [36]. In this context of incidental findings, the GfH recommends that results should only be returned to the individual concerned if there is a relevant risk of a genetic disease or if an effective treatment or preventive measures exist [36, 37]. In addition, the German Genetic Diagnostics Act requires anyone who is aware of a genetic diagnosis pertaining to them to inform the insurance company of this before taking out a new insurance policy. This emphasizes the differences between standard lab test results and genetic findings and further explains why healthcare professionals may handle these findings with a certain degree of exceptionalism and paternalism.

Interestingly, within our PRAEGNANT study, the majority of research results (64.3%) were not validated before their return, even though the accompanying cover letter clearly recommended validation. Correct assignment of results to sample and patient data cannot be guaranteed due to multiple pseudonymization processes and multiple handling of samples (blood and DNA) during processing and analysis. Errors can occur during sample tracking, labeling, and processing at the study center, the central biobank unit, or the associated analysis laboratory [27]. Any mis-assignment of results to an individual could cause harm to the patient if results are not validated beforehand. In particular, clinical decisions should not be based on research results alone. But even where there are no clinical consequences to the results, patients may be negatively affected by the disclosure of non-validated findings due to anxiety and insecurity, on their part or on that of relatives. The ACMG policy statement on reporting of incidental genomic findings recommends that physicians should provide comprehensive pre- and post-test counseling to patients, including the consultation of a clinical geneticist for ordering, interpretation, and communication of genomic testing [27]. In all cases reported via our questionnaire, validation of genetic research results was fully covered by the patient’s health insurance, meaning that financial resources did not present an obstacle to initiating validation. Taking this into account, additional training of physicians in this area may be necessary.

However, within our project, three physicians reported that research results were validated after disclosure of genetic findings to the patients. This may be the case for a larger number of patients, and some genetic research findings may be validated during the later progression of the disease, if, for example, a change of therapy (e.g., PARPi treatment) is indicated. This scenario was not part of the questionnaire, but could be of interest for further follow-up surveys.

In summary, our results provide novel insights into the return of genomic research results within a German registry study. Limitations of our study include the fact that not all physicians provided information on whether patients were aware of germline mutations beforehand. This was particularly the case within the subgroup of patients that did not receive results. For 40 out of 89 cases, information about knowledge of mutations was missing, meaning that the percentage presented here may be biased for this subgroup. Further, our questionnaire did not ask about validation of genetic findings after disclosure to patients; this means that the data can only be interpreted with regard to validation of results before return to the patients. In addition, influence on benefit to the patient and quality of life was assessed by physicians themselves. A clearer picture regarding patients’ point of view could be obtained by surveying the patients directly.

## Supplementary Information

Below is the link to the electronic supplementary material.

Supplementary file1 (DOCX 26 KB)

Supplementary file2 (DOCX 49 KB)

## Data Availability

The datasets generated and/or analyzed during the current study are not publicly available due to GDPR regulations, but are available from the corresponding author on reasonable request.
