# Supplementary material for: Return of individual genomic research results within the PRAEGNANT multicenter registry study
Source: Breast Cancer Res Treat. 2022 Nov 21;197(2):355–68. doi: 10.1007/s10549-022-06795-x (PMC9822879; doi:10.1007/s10549-022-06795-x)
Supplement: Supplementary file 1 — Supplementary file1 (DOCX 26 KB) [file 10549_2022_6795_MOESM1_ESM.docx]

Supplementary Table 1

| Age range at diagnosis (yrs) | Mol. Subtype | Gene* | Known mutation | Reason for not returning | Other reason |
| --- | --- | --- | --- | --- | --- |
| >50 | HER2 positive | **ERCC2** | **No, none** | **No relevance** |  |
| >50 | TNBC | BRCA1 | Yes, all | No consent at study inclusion & No relevance | Known mutation |
| >50 | Luminal A | **MUTYH**; PPM1D** | **No, none** | **No relevance** |  |
| >50 | TNBC | BRCA1 | No, none | No relevance | Known mutation |
| >50 | HER2 positive | **MUTYH**** | **No, none** | **No relevance** |  |
| >50 | TNBC | **TP53**** | **No, none** | **No relevance & Other reason** | **No relevance** |
| >50 | Luminal A | **PPM1D** | **No, none** | **No relevance & Other reason** | **No relevance** |
| >50 | HER2 positive | NBN |  | No relevance & Other reason | No relevance |
| >50 | - | **RINT1** | **No, none** | **No relevance & Other reason** | **No relevance** |
| >50 | Luminal B, HER2 negative | ATM |  | No consent at inclusion in study |  |
| >50 | Luminal A | BRCA1; CHEK2 |  | No consent at inclusion in study |  |
| >50 | Luminal A | **PMS2**** | **No, none** | **No relevance** |  |
| >50 | Luminal A | **PPM1D** | **No, none** | **No relevance** |  |
| >50 | Luminal A | BRCA2 | Yes, all | Other reason | Known mutation |
| >50 | Luminal A | **EPCAM** | **No, none** | **No relevance** |  |
| >50 | TNBC | ERCC2 |  | No consent at inclusion in study |  |
| >50 | TNBC | BRCA1 | Yes, all | Other reason | Known mutation |
| >50 | Luminal A | ATM |  | Other reason | Consent withdrawn |
| >50 | Luminal A | ERCC2 |  | Other reason | Consent withdrawn |
| >50 | Luminal B, HER2 negative | PALB2 |  | Other reason | Lost to follow-up |
| >50 | Luminal A | BRCA2 |  | Other reason | Consent withdrawn |
| >50 | Luminal A | ATM |  | Other reason | Consent withdrawn |
| >50 | Luminal B, HER2 negative | CHEK2 |  | Other reason | Lost to follow-up |
| >50 |  | PALB2; CHECK2 |  | Other reason | Consent withdrawn |
| >50 | Luminal A | ATM |  | No consent at inclusion in study |  |
| >50 | Luminal B, HER2 negative | BRCA2; CHEK2 |  | Other reason | Lost to follow-up |
| >50 |  | RECQL |  | No relevance |  |
| >50 | Luminal B, HER2 negative | PPM1D |  | Other reason | Lost to follow-up |
| >50 | Luminal A | NBN |  | Other reason | Lost to follow-up |
| >50 | Luminal A | CHEK2 | No, none | No consent at inclusion in study |  |
| >50 | Luminal A | NBN | No, none | Other reason | Lost to follow-up |
| >50 | - | CHEK2; FANCM |  | Other reason | Lost to follow-up |
| >50 | - | PALB2 |  | Other reason | Lost to follow-up |
| >50 | HER2 positive | NBN |  | Other reason | Lost to follow-up |
| >50 | - | **CHEK2** | **No, none** | **No relevance & Other reason** | No therapeutic relevance |
| >50 | Luminal A | XRCC2 |  | Other reason | Lost to follow-up |
| >50 | Luminal B, HER2 negative | BLM |  | Other reason | Consent withdrawn |
| >50 | Luminal A | ATM |  | Other reason | Consent withdrawn |
| 18-50 | Luminal A | BRCA2; APC | Yes, but not all | Other reason | Clinical diagnostics not yet performed |
| 18-50 | Luminal A | BRCA2 | Yes, all | No relevance | Known mutation |
| 18-50 | Luminal A | BRCA2; MUTYH | Yes, but not all | No relevance | Known mutation |
| 18-50 | Luminal A | PALB2 | Yes, all | No relevance | Known mutation |
| 18-50 | Luminal B, HER2 negative | BRCA1; ERCC2 | Yes, but not all | No relevance | No relevance & known mutation |
| 18-50 | HER2 positive | **FANCM** | **No, none** | **No relevance & Other reason** | No breast cancer panel gene |
| 18-50 | Luminal A | **FANCM** | **No, none** | **Other reason** | No breast cancer panel gene |
| 18-50 | Luminal A | BRCA2; CHEK2 | Yes, all | No relevance | Known mutation |
| 18-50 | - | BRCA1 | Yes, all | No relevance | Known mutation |
| 18-50 | - | BRCA1; MUTYH | Yes, all | No relevance | Known mutation |
| 18-50 | Luminal B, HER2 negative | **PALB2** | **No, none** |  |  |
| 18-50 | Luminal A | **ERCC2** | **No, none** | **No relevance** |  |
| 18-50 | HER2 positive | **CHEK2** | **No, none** | **No relevance & Other reason** | **No relevance** |
| 18-50 | Luminal A | BRCA2 | Yes, all | No relevance & Other reason | Known mutation |
| 18-50 | - | **MUTYH**** | **No, none** | **No relevance & Other reason** | **No relevance** |
| 18-50 | - | **ERCC2** | **No, none** | **No relevance & Other reason** | **No relevance** |
| 18-50 | TNBC | BRCA1 | Yes, all | Other reason | Known mutation |
| 18-50 | - | BRCA1 | Yes, all | Other reason | Known mutation |
| 18-50 | Luminal A | CHEK2 |  | No consent at inclusion in study |  |
| 18-50 | Luminal A | BRCA1 | Yes, all | Other reason | Known mutation |
| 18-50 | TNBC | BRCA1 | Yes, all | Other reason | Known mutation |
| 18-50 | Luminal A | PALB2 |  | Other reason | Known mutation |
| 18-50 | - | **RECQL** | **No, none** | **No relevance** |  |
| 18-50 | Luminal B, HER2 negative | NBN |  | No relevance |  |
| 18-50 | - | BRCA1 | Yes, all | Other reason | Known mutation |
| 18-50 | Luminal A | MUTYH | Yes, but not all | No relevance |  |
| 18-50 | Luminal A | PALB2 |  | No relevance |  |
| 18-50 | Luminal B, HER2 negative | TP53 | Yes, all | Other reason | Known mutation |
| 18-50 | Luminal A | BRCA2 | Yes, all | Other reason | Known mutation |
| 18-50 | Luminal A | MUTYH | Yes, all | No relevance |  |
| 18-50 | - | FANCC | Yes, all | Other reason | Known mutation |
| 18-50 | Luminal B, HER2 negative | BRCA1 | Yes, all | Other reason | Known mutation |
| 18-50 | - | BRCA1 | Yes, all | Other reason | Known mutation |
| 18-50 | Luminal B, HER2 negative | **MSH6**** | **No, none** | **No relevance** |  |
| 18-50 | TNBC | BRCA1 |  | Other reason | Consent withdrawn |
| 18-50 | Luminal A | ATM |  | Other reason | Consent withdrawn |
| 18-50 | HER2 positive | CHEK2 |  | Other reason | Consent withdrawn |
| 18-50 | Luminal B, HER2 negative | CHEK2 |  | Other reason | Lost to follow-up |
| 18-50 | Luminal A | ERCC3 |  | Other reason | Lost to follow-up |
| 18-50 | Luminal A | NBN |  | Other reason | Lost to follow-up |
| 18-50 | Luminal A | PALB2 |  | Other reason | Lost to follow-up |
| 18-50 | Luminal B, HER2 negative | BRCA1; PPM1D |  | Other reason | Lost to follow-up |
| 18-50 | TNBC | BRCA1 | Yes, all | Other reason | Known mutation |
| 18-50 | - | CHEK2 |  | Other reason | Lost to follow-up |
| 18-50 | HER2 positive | CHEK2; APC |  | Other reason | Consent withdrawn |
| 18-50 | TNBC | BRIP1 |  | Other reason | Lost to follow-up |
| 18-50 | - | FANCC |  | Other reason | Not requested |
| 18-50 | HER2 positive | **MUTYH**** | **No, none** | **No relevance** |  |
| 18-50 | HER2 positive | RAD51D | No, none | No consent at inclusion in study |  |
| 18-50 | HER2 positive | CHEK2 |  | Other reason | Consent withdrawn |
| 18-50 | Luminal A | BRCA2; RECQL |  | No consent at inclusion in study |  |

* Genes in bold type are those for which mutations were detected that were not known of by the patient but classified as of “no relevance” by the physician.

** These findings were not returned to the patient, not known of by the patient and classified as of “no relevance” by the physician, but these genes are included on the “minimum list” as defined in the statement issued by the American College of Medical Genetics and Genomics (Kalia et al., 2017).
