# Supplementary material for: Return of individual genomic research results within the PRAEGNANT multicenter registry study
Source: Breast Cancer Res Treat. 2022 Nov 21;197(2):355–68. doi: 10.1007/s10549-022-06795-x (PMC9822879; doi:10.1007/s10549-022-06795-x)
Supplement: Supplementary file 2 — Supplementary file2 (DOCX 49 KB) [file 10549_2022_6795_MOESM2_ESM.docx]

| **1.00)** | PRAEGNANT patient ID:**«PRAEGNANT_PatID»** | | PRAEGNANT study site ID:**«PRAEGNANTstudysite»** | | | | |
| --- | --- | --- | --- | --- | --- | --- | --- |
| **1.01)** | Did you communicate the results of the PRAEGNANT test to the patient? | Yes |  | | | | No |
| **1.02)** | If you did NOT inform the patient, what were your reasons for this?  (Multiple answers possible) | The patient declined to be informed of the results upon enrolment in the study  The patient declined to be informed of the results after consultation  The results are not relevant to the patient or their family  I do not trust the results  I did not understand the results  No mutations were detected  Other reason: ________________________ | | | | | |
| **1.03)** | Were the mutations detected in the PRAEGNANT test already known to the patient? | Yes, all | Yes, but not all | | | | No, none |
| **1.04)** | If the results were communicated to the patient, were they validated beforehand? | Yes, all | Yes, but not all | | | | No, none |
| **1.05)** | If results were validated, which mutations were analyzed? | All mutations detected  Only mutations selected by me  Only mutations selected by the geneticist | | | | | |
| **1.06)** | If only selected mutations were validated, which ones were they? | _________________________________________  _________________________________________ | | | | | |
| **1.07)** | If results were validated, were they in line with the PRAEGNANT research results? | Yes, all of them | |  | No, not ___ (number of  *mutations*) | | |
| **1.08)** | If the results from the PRAEGNANT test differed from the validated ones, please specify which mutations were different: | PRAEGNANT germline mutation  _______________ _______________ | | | | Clinically validated germline mutation  _______________ _______________ | |
| **1.09)** | Did the patient’s health insurance cover the cost of validation of the test results? | Yes |  | | | | No |
|  |  | If no, what was/were the reason(s) for this? ____________________ | | | | | |
| **1.10)** | Did the results of the PRAEGNANT test influence current or future treatment decisions? | Yes |  | | | | No |
|  |  | If yes, what was/were the reason(s) for this? _______________________ | | | | | |
| **1.11)** | Did the results of the PRAEGNANT test have any influence on whether relatives will be genetically tested? | Yes | I do not know | | | | No |
| **1.12)** | Do you think the patient benefited from the results of the PRAEGNANT germline testing? | Yes |  | | | | No |
|  |  | Reason: ______________________________ | | | | | |
| **1.13)** | Do you think the results will affect your patient's quality of life? | Yes, positively | No | | | | Yes, negatively |
|  |  | Reason: ______________________________ | | | | | |
